# Evaluation of Patterns of Trauma Reporting to the Emergency Department During the First COVID-19 Lockdown in India

**DOI:** 10.7759/cureus.14609

**Published:** 2021-04-21

**Authors:** Swagat Mahapatra, Shiv Shanker Tripathi, Vineet Kumar, Suruchi Ambasta, Anurag Agarwal, Rajiv Ratan Singh Yadav, Divyansh Krishna

**Affiliations:** 1 Orthopedic Surgery, Dr. Ram Manohar Lohia (RML) Institute of Medical Sciences, Lucknow, IND; 2 Emergency Medicine, Dr. Ram Manohar Lohia (RML) Institute of Medical Sciences, Lucknow, IND; 3 Anesthesiology, Sanjay Gandhi Postgraduate Institute of Medical Sciences, Lucknow, IND; 4 Anesthesia, Critical Care, and Pain Medicine, Dr. Ram Manohar Lohia (RML) Institute of Medical Sciences, Lucknow, IND; 5 Emergency Medicine, Belgavi Institute of Medical Sciences, Belgaum, IND

**Keywords:** trauma, covid-19, lockdown, demography, alcohol, emergency: india

## Abstract

Background

On March 24, 2020, the Government of India declared a nationwide lockdown and a series of measures aimed at limiting the spread of the coronavirus disease 2019 (COVID-19) infection. This led to dynamic changes in patient inflow and management in the emergency department.This study aims to evaluate the impact of the pre-lockdown and lockdown periods on the demography of trauma in a tertiary care teaching hospitaland to compare it with the homologous period of 2019.

Methods

The trauma caseloads between March 25, 2020, and April 14, 2020, and that of the homologous period of 2019 were thoroughly analyzed and compared retrospectively.

Results

There was an overall decrease in trauma patients. Elderly male patients had an increased incidence of injury during the lockdown period with a significant p-value (0.0009). There was a significant increase in the number of minor orthopedic procedures while there was a significant decrease in the number of major orthopedic procedures. Fractures of the proximal femur were significantly increased during the lockdown period (p-value0.011) and fractures of the femur and tibia shaft were significantly decreased during the lockdown period (p-value0.002). Fractures of the distal radius were significantly increased during the lockdown period (p-value0.005) and fractures of the shaft of humerus, radius, and ulna were significantly decreased during the lockdown period (p-value0.028). Injuries following fall, trivial trauma, and animal-induced trauma were significantly increased (p-values <0.0001, <0.0001, 0.014, respectively), whereas injuries following sports and motor vehicle accidents were significantly decreased (p-value 0.006, <0.0001, respectively). The number of patients reaching within the golden hour (<1 hour) was significantly increased (p-value 0.0003). The total number of patients presenting under the influence of alcohol during the lockdown period was significantly decreased (p-value- <0.0001). The use of government-sponsored ambulances for transport to the hospital was significantly increased during the lockdown period (p <0.0001).

Conclusion

Strict administrative measures had a high impact on the number and epidemiology of trauma with remarkable changes. There was a decreased number of trauma cases but the mechanism, type, and management of these cases were significantly altered from the homologous period of the previous year.

## Introduction

The novel coronavirus disease 2019 (COVID-19) first reported in China in December 2019 has rapidly spread across the entire world. The first case of COVID-19 was detected in India on January 30, 2020. The World Health Organization (WHO) declared the COVID-19 outbreak as a global pandemic on March 11, 2020. The Government of India invoked the Epidemic Disease Act, 1987, and declared COVID-19 an epidemic of serious concern. On March 24, 2020, the Government of India declared a nationwide lockdownand a series of measures, including stoppage of vehicular movements, aimed at limiting the spread of the COVID-19 infection [1]

The plethora of measures initiated to arrest the spread of the virus included restricting all non-essential travel, prohibiting the sale of alcohol, closure of workplaces for nonessential services, and restricting social gatherings. Consequently, all vehicular movement, schools, construction work, industries, and national highways were shut down.Essential food supplies and medical needs were arranged without much movement outside.

Many people have altered their daily routines in response to the novel coronavirus outbreaks, and these changes had brought about atypical trends in injuries related to trauma. There has been a sharp and noticeable decline in the number of acute trauma referrals, admissions, operations, and aerosolizing anesthetic procedures in the emergency department [2]. As a result of the COVID-19 pandemic, trauma trends have drastically changed. COVID-19 also witnessed alterations in medical treatment protocols and during the lockdown, all elective surgeries were canceled across the country except the lifesaving ones. This led to a significant fall in elective cases and only trauma and emergency cases were accepted in all hospitals.

Despite the restriction on mobility of people and vehicles, sporadic trauma cases continued to arrive at most emergency departments, in small numbers. This study brings a comparative analysis of the effect of this complete lockdown on the pattern of orthopedic workload and trauma with similar, normal periods in previous years. There is little clinical data available in our country about the patterns and volumes of injury that can impact hospital resources and manpower during periods of strict community lockdowns. This being a unique and new situation, hospital administratorscontinueto be baffled regarding the optimum utilization of resources in managing non-COVID trauma patients requiring acute care [3]. Little is known and reported about the patterns and volumes of injury that can impacthospital resources during periods of community lockdowns in such pandemic situations, which is totally unprecedented in nature [4].

The aim of this study was to understand the effect of the lockdown and reduced vehicular and industrial activity on the pattern and number of orthopedic trauma cases. The secondary objective was to study epidemiology, patterns of injuries, mechanisms, and causes of the same. The study may help predict the pattern of orthopedic injuries that may arise in the future, in a post-pandemic world where there will be a change in the way we all live.

## Materials and methods

This study was conducted at an 1100-bedded tertiary care multispecialty teaching hospital. This was a retrospective observational study conducted in the department of emergency medicine after obtaining institutional ethics committee approval (IEC number- 102/20). All hemodynamically stable trauma patients, from five years to 80 years, visiting our emergency during the national lockdown-1 period (March 25, 2020, to April 14, 2020) were included.Pregnant patients and patients with major life-threatening co-morbidities were excluded from our study.Data from records and case sheets of trauma patients during this period and the same period of the previous year (March 25, 2019 - April 14, 2019) were obtained. Epidemiological data were collected, including age, sex, mode of injury, alcohol status, and time of injury, time delay in presentation to the emergency, mode of transfer (personal/hired vehicle or 108 ambulances). Data regarding the type and location of the fracture and bony and soft tissue procedures performed were also collected and tabulated. Detailed tabulation of data was done and appropriate statistical analysis was performed.

Data analysis

Continuous data were analyzed as mean standard deviation. Categorical data were reported as numbers and percentages and were analyzed using the Chi-square test or Fishers exact test as appropriate. The value p<0.05 was considered statistically significant.

## Results

In our series, the total number of patients reporting to the emergency department in the pre-lockdown time frame was 278, with 173 (62.2%) being males and 105 (37.8%) being females. During the lockdown period, the total number of patients presenting was 216, with 121 (56%) being males and 95 (44%) being females. Elderly male patients had an increased incidence of injury during the lockdown period with a significant p-value (0.0009). The rest of the age groups showed no significant difference in presentation (Table 1).

**Table 1:** Age and gender-wise distribution of cases reporting at the trauma emergency pre-lockdown and during lockdown

Gender and Age Group	Male		Chi-square	p-value	Female		Chi-square	p-value
	Pre-Lockdown (n=173)	During Lockdown (n=121)			Pre-Lockdown (n=105)	During Lockdown (n=95)		
Paediatric (0-16)	21 (12%)	13 (10.7%)	0.0334	0.854	12 (11.4%)	10 (10.5%)	0.00	1.00
Young adult (17-25)	53 (30.6%)	31 (18%)	0.649	0.42	31 (29.5%)	27 (28.4%)	0.0002	0.987
Adult (26-60)	67 (38.7%)	33 (`27.2%)	3.668	0.055	38 (36.2%)	29 (30.5%)	0.4865	0.4854
Elderly (>60)	32 (18.5%)	44 (36.4%)	10.94	0.0009	24 (22.8 %)	29 (30.5%)	1.138	0.286

Soft tissue procedures, including laceration suturing, wound debridement, and tendon repairs, performed in the emergency department were 33 pre-lockdown and 45 during the lockdown. These procedures increased during the lockdown period but were statistically nonsignificant in comparison to pre-lockdown values (Table 2).

**Table 2:** Soft tissue procedures performed pre-lockdown and during the lockdown

Procedure	Pre-lockdown n=33	During lockdown n=45	Chi-square	p-value
Laceration suturing	21 (63.6%)	29 (64%)	0.000	1.000
Wound debridement	7 (21%)	8 (18%)	0.008	0.989
Tendon repair	5 (15%)	8 (18%)	0.000	1.000

The total number of orthopedic procedures performed pre-lockdown was 128.92 (72%) were major cases and 36 (28%) were minor cases. During the lockdown period, the total number of orthopedic procedures performed was 70, with 17 (24%) being major and 53 (76%) being minor.This difference was significant with p-values of <0.0001. There was a significant increase in the number of minor procedures while there was a significant decrease in the number of major procedures (Table 3).

**Table 3:** Orthopedic procedures performed pre-lockdown and during the lockdown

Orthopedic surgeries	Pre-lockdown n=128	During lockdown n=70	Chi-square	p-value
Major	92 (72%)	17 (24%)	39.51	<0.0001
Minor	36 (28%)	53 (76%)		

Fractures sustained in the lower limb were divided into subgroups of fractures of the proximal femur, fractures around the knee, fractures of the femur and tibia shaft, and fractures of the foot and ankle. The total number of lower limb fractures presenting pre-lockdown was 118 and during lockdown were 70. Among the subgroups, fractures of the proximal femur were significantly increased during the lockdown period (p-value0.011) and fractures of the femur and tibia shaft were significantly decreased during the lockdown period (p-value0.002). The rest of the lower limb fractures did not show a significant change. Fractures sustained in the upper limb were divided into subgroups of fractures of the clavicle, scapula, and proximal humerus, fractures around the elbow, fractures of the shaft of the humerus, radius and ulna, fractures of the distal radius, and fractures of the wrist and hand. The total number of upper limb fractures presenting pre-lockdown was 73 and during lockdown were 68. Among the subgroups, fractures of the distal radius were significantly increased during the lockdown period (p-value0.005) and fractures of the shaft of the humerus, radius, and ulna were significantly decreased during the lockdown period (p-value- 0.028). The rest of the upper limb fractures did not show a significant change. Fractures sustained in the spine and pelvis were divided into subgroups of cervical spine fractures, fractures of the dorsolumbar spine, andfractures of the pelvis and acetabulum. The total number of spine and pelvic fractures presenting pre-lockdown was 21 and 10 during the lockdown. Among the subgroups, cervical spine, dorsolumbar spine, and pelvis and acetabulum fractures did not show a significant change. Pediatric fractures including both upper and lower limbs did not show any significant change during the lockdown period (p-value0.236) (Table 4).

**Table 4:** Site of fracture among cases reporting pre-lockdown and during the lockdown

Site of Fracture	Pre-lockdown	During lockdown	Chi-square	p-value
Lower limb	(n=118)	(n=70)		
Proximal femur	42 (36%)	39 (56%)	6.456	0.011
Fractures around knee	22 (18.6%)	7 (10%)	1.897	0.168
Femur & Tibial shaft	37 (31.4%)	7 (10%)	10.018	0.002
Foot & Ankle	17 (14%)	17(24%)	2.266	0.132
Upper limb	(n=73)	(n=68)		
Clavicle, Scapula, & Proximal humerus	7 (10%)	4 (6%)	0.2559	0.613
Fractures around the elbow	18 (25%)	11 (16%)	1.074	0.299
Shaft of Humerus, Radius, & Ulna	19 (26%)	7 (10.3%)	4.796	0.028
Distal radius	16 (22%)	31 (46%)	7.8432	0.005
Wrist & Hand	13 (18%)	15 (22%)	0.1772	0.673
Spinal fractures	(n=21)	(n=10)		
Cervical spine	7 (n=33%)	5 (50%)	0.246	0.619
Dorsolumbar spine	8 (38%)	4 (40%)	0.000	1.00
Pelvis and acetabulum	6 (29%)	1 (10%)	0.485	0.486
Pediatric fractures	(n=33)	(n=23)		
Upper limb	23 (70%)	20 (87%)	1.4003	0.236
Lower limb	10 (30%)	3 (13%)		

The mechanism of injury of cases presenting pre- and during lockdown was subdivided into fall, sports injuries, motor vehicle accidents, gunshot injuries, animal-induced, and injuries following trivial trauma. Injuries following fall, trivial trauma, and animal-induced trauma were significantly increased (p-values <0.0001, <0.0001, 0.014, respectively), whereas injuries following sports and motor vehicle accidents were significantly decreased (p-value 0.006, <0.0001, respectively). Injuries following a gunshot wound were nonsignificantly decreased (p-value0.352) (Table 5).

**Table 5:** Mechanism of injury among cases reporting pre-lockdown and during the lockdown

Mechanism of injury	Pre-lockdown (n=278)	During lockdown (n=216)	Chi-square	p-value
Fall	79 (28%)	111 (51%)	26.138	<0.0001
Sports	39 (14%)	13 (6%)	7.452	0.006
Motor vehicle accident	117 (42%)	21 (10%)	61.647	<0.0001
Gunshot	5 (2%)	1 (0.5%)	0.865	0.352
Animal induced	9 (3%)	19 (9%)	6.024	0.014
Trivial trauma	29 (10%)	52 (24%)	15.52	<0.0001

Time elapsed between trauma and arrival in the emergency department was noted pre-lockdown and during the lockdown. The number of patients reaching within the golden hour (<1 hour) was significantly increased (p-value 0.0003). The total number of patients presenting under the influence of alcohol during the lockdown period was significantly decreased (p-value<0.0001). The use of government-sponsored ambulances for transport to the hospital was significantly increased during the lockdown period (p <0.0001) (Table 6).

**Table 6:** Presentation of cases reporting pre-lockdown and during the lockdown

Presentation at emergency	Pre-lockdown (n=278)	During lockdown (n=216)	Chi-square	p-value
Delay in presentation to Hospital				
<1 hour	85 (31%)	101 (47%)	12.88	0.0003
>1 hour	193 (69%)	115 (53%)		
Influence of alcohol				
Under influence	56 (20%)	3 (1%)	38.89	<0.0001
Not under influence	222 (80%)	213 (99%)		
Mode of transport to hospital				
Hired/self vehicle	112 (40%)	43 (20%)	22.511	<0.0001
Govt.-sponsored ambulance	166 (60%)	173 (80%)		

## Discussion

The total number of trauma cases presenting during the lockdown period was significantly decreased, similar to findings by Hazra et al. [5]. Elderly male patients had an increased incidence of injury during this period. This may be explained by the fact that due to lockdown measures, all movement outside the house was completely curtailed and exposure to sunlight was also decreased, predisposing to vitamin D deficiency [6]. Also, elders had to take part in daily household chores due to a lack of household help. This could be the cause of increased fragility in elderly males. Elderly females, on the other hand, are actively involved in housework in our society, with limited outside movement. Hence, there was no great change for them even in the lockdown period. Pediatric upper limb trauma was increased while lower limb trauma was decreased but was not statistically significant. Upper extremity fractures usually occur in children following a fall during play while lower limb fractures occur following a high-velocity injury. Due to forced school leaves, children were home all the time and were engaged in games at home, which led to frequent falls and injuries of the upper extremity. There was no other statistically significant change in injury pattern on an age and sex basis.

Fractures around the hip and fractures of the distal radius, which are fragility fractures encountered in the elderly, were found to be significantly increased [7]. This may again be explained by the fact that the elderly were involved in unaccustomed household work due to the absence of household help during the lockdown phase. Also, the lack of exercise and sunlight exposure increased the chances of fragility fractures even on trivial trauma [6].

Fractures of long bones, including the femur, tibia, humerus, radius, and ulna, were significantly decreased. This may be explained by the fact that these injuries usually occur following high-velocity trauma in the form of motor vehicle accidents [8]. Due to the complete shutdown and decrease in road traffic accidents, these fractures were decreased.

Fractures of the axial skeleton, including spine and pelvis injuries, usually occur following high-velocity injuries or following fall from heights. Although road traffic accidents were decreased, injuries following a fall were significantly increased in our series. This led to a nonsignificant difference in the number of these fractures during the lockdown.

Injuries following motor vehicle accidents and sports injuries were decreased due to lack of traffic and vehicular movements and complete stoppage of outdoor sports activities. Injuries following falls and trivial trauma were significantly increased. This may be explained by a longer stay at home and exposure to unaccustomed household chores. Animal-induced trauma was also found to be significantly increased. In our society, stray dogs and animals usually feed themselves from food wastes and dustbins. During the lockdown period, there was a complete stoppage of the availability of food wasteon the roads. This may have led to aggressive behavior and unprovoked attacks of these strays, causing trauma. This was in contrast to a study by Morris et al. who reported no change in dog bites and other animal-induced trauma [9].

There was a non-significant decrease in episodes of firearm injury. This was probably due to the implementation of a curfew-like situation and strict vigilance by the administration and police personnel. This was in contrast to reports by Abdallah et al. who reported increased firearm injury during the pandemic lockdown [10].

Due to the involvement of people in new and unfamiliar types of work, there was an increase in soft tissue injuries requiring minor soft tissue operative procedures.Males involved in kitchen work, gardening, use of sharp instruments for repairing minor household appliances, and involvement of factory workers in farming at their hometown are a few examples of unaccustomed work. There was an increase in minor orthopedic procedures due to the same reasons mentioned above. However, there was a significant decrease in major orthopedic and elective orthopedic procedures during the lockdown period. This was probably due to a decrease in overall trauma and fractures requiring surgical interventions. Elective procedures were postponed due to safety protocols as per COVID guidelines, as well as the redistribution of health personnel for the care of COVID patients. These findings were similar to the study by Ribau et al. [11].

The use of government-sponsored ambulances was significantly increased due to the lack of availability of private vehicles, as well as the difficulty in obtaining clearance for road travel across borders. Also, there was excellent availability of ambulances due to administrative vigilance. Maximum patients reached emergency within one hour of trauma. This was possible due to the complete lack of traffic on the roads and the use of government ambulances. Government ambulances also have the advantage of being Global Positioning System (GPS) enabled and drivers are experienced regarding the type of services offered by hospitals [12].

Alcohol intake promotes risk-taking behavior and predisposes to accidents [13]. Patients sustaining trauma under alcohol influence were significantly decreased due to the non-availability of alcohol during the lockdown. This was similar to the findings of Manyoni et al. [14].The present study is limited by its focus on one isolated emergency department and the lack of inclusion of other regional trauma centers.

## Conclusions

The COVID-19 pandemic has had a significant impact on trauma and emergency caseloadsand services. The administrative measures during the lockdown significantly affected the epidemiology and the total number of trauma patients. There was a reduction of accidental, work-related, and sports-related trauma but there was an increase in fragility fractures. The vigilant administrative efforts, however, led to quicker response to the trauma patients and a significant decrease in injuries under the influence of alcohol. These findings will not only serve as a record of this particular pandemic lockdown but, with deeper analysis and more extensive studies, may help in developing injury prevention and prompt emergency management strategies.
